# The effects of water temperature on the juvenile performance of two tropical damselfishes expatriating to temperate reefs

**DOI:** 10.1038/s41598-019-50303-z

**Published:** 2019-09-26

**Authors:** Lucas D. Djurichkovic, Jennifer M. Donelson, Ashley M. Fowler, David A. Feary, David J. Booth

**Affiliations:** 1 School of Life Sciences, University of Technology, Sydney, PO Box 123, Broadway, New South Wales 2007 Australia; 2 ARC Centre of Excellence for Coral Reef Studies, James Cook University, Townsville, QLD 4811 Australia; 3 New South Wales Department of Primary Industries, Sydney Institute of Marine Science, Mosman, NSW 2088 Australia; 4 MRAG Ltd., 18 Queen Street, London, W1J 5PN United Kingdom

**Keywords:** Animal migration, Marine biology

## Abstract

Ocean warming associated with global climate change is already inducing geographic range shifts of marine species. Juvenile coral reef fishes transported into temperate latitudes (termed ‘vagrant’ fishes) can experience winter water temperatures below their normal thermal minimum. Such environmental extremes may increase energetic costs for such fishes, resulting in reduced performance, which may be the governing factor that limits the potential for poleward range expansion of such fishes. This study compared the juvenile physiological performance and behaviour of two congeneric tropical damselfishes which settle during austral summer months within temperate eastern Australia: *Abudefduf vaigiensis* have an extended southern range, and lower threshold survival temperature than the congeneric *A. whitleyi*. Physiological and behavioural performance parameters that may be affected by cooler temperature regimes at higher latitudes were measured in aquaria. Lower water temperature resulted in reduced growth rates, feeding rates, burst escape speed and metabolic rates of both species, with significantly reduced performance (up to six-fold reductions) for fishes reared at 18 °C relative to 22 °C and 26 °C. However, *A. whitleyi* exhibited lower growth rates than *A. vaigiensis* across all temperatures, and lower aerobic capacity at the lowest temperature (18 °C). This difference between species in growth and metabolic capacity suggests that the extended southern distribution and greater overwintering success of *A. vaigiensis*, in comparison to *A. whitleyi* is related to thermal performance parameters which are critical in maintaining individual health and survival. Our results support previous findings in the region that water temperature below 22 °C represents a critical physiological threshold for tropical *Abudefduf* species expatriating into temperate south-eastern Australia.

## Introduction

The geographic ranges of species are determined by their ability to successfully disperse into environments and then withstand local conditions^1,2^. A species’ range can be controlled by a combination of both climatic (e.g. local temperature, levels of precipitation and extreme weather events^3,4^) and ecological factors (e.g. competition for suitable food and resources^5^). Near the periphery of a species’ range, conditions may become increasingly stressful, either due to reductions in suitable resources and/or reductions in physiological suitability^6^. For range restrictions related to thermal physiology, climate change threatens to alter the distribution of organisms globally by shifting them towards cooler climates in a phenomenon referred to as poleward range expansions or ‘range shift’^7–9^. Range shifts have already been documented for numerous populations of marine organisms^10,11^ and many of these shifts are occurring faster than the global average of all environments^12^.

Successful range shifts require the ability for species to disperse to new locations. The majority of marine organisms, including fishes, possess a dispersive life phase during larval development^13,14^. This allows local or regional currents to potentially facilitate poleward range expansion, including the expatriation of tropical fish species into temperate habitats^15^. For example, the East Australian Current (EAC) is a western boundary current that transports the larvae of tropical reef fish polewards to temperate environments of Australia’s south-east (SE) coastline, with juveniles of many coral-reef species already observed inhabiting temperate rocky reefs within austral summer months^16,17^. Range shift capacity of species is currently limited due to high mortality during the cooler winter months^18^. However, with increased ocean warming and strengthening of the EAC associated with climate change^19^, it is likely that the occurrence and persistence of expatriated tropical species in this region will increase in the future associated with higher water temperatures within winter months^18^.

Warming of SE Australian waters has already increased the abundance and diversity of new settlers of a range tropical fishes into temperate habitats, with 47 species recorded during surveys between 2003–2005^16^ and an additional 22 species observed in a study conducted 10 years later^17^. Species from the damselfish genus *Abudefduf* have been one of the most commonly observed vagrants, in some locations being observed on 50% of surveys during recruitment months^16^. From January to May, 5 species of *Abudefduf* spp. vagrants settle into temperate rocky reef habitats along the New South Wales (NSW) coast^16,20,21^. The onset of winter results in almost 100% mortality among these tropical fishes when water temperature drops below 17 °C^18^, therefore over-winter survival appears to be the major limitation to the poleward range expansion of these tropical vagrants into temperate Australia. Previous research has shown that juvenile abundance of these tropical vagrants on temperate reefs varies greatly, not just between unrelated species but also between congenerics^14,16^. For example, *A. vaigiensis, A. sexfaciatus and A. bengalensis* are found more frequently, abundantly and further south in SE Australia than *A. whitleyi*, which has only been observed at a few locations sporadically and have rarely been recorded at latitudes higher than 37°^16,17^. Over-winter survival also differs among *Abudefduf* species, with previous modelling indicating a warmer threshold survival temperature for vagrant *A. whitleyi* (16.5–16.8 °C) relative to both *A. vaigiensis* and *A. bengalensis* (12.8–13 °C), and no overwintering recorded for *A. sexfasciatus* in temperate SE Australia^18^. It is predicted by the year 2080 that the overwinter survival of tropical vagrants in this region will occur annually due to ocean warming above minimum thermal thresholds^18^; therefore, it is important to understand which species will most likely successfully recruit and sustain populations.

Both physiological and behavioural performance of marine fishes are linked to ambient thermal conditions^22,23^. Ectotherm metabolic theory predicts that temperatures close to critical thermal maxima or minima will negatively impact kinetic activity and destabilise heat-shock proteins^24,25^. When approaching the thermal limits, physiological constraints also occur through reduced aerobic capacity due to limitations of the circulatory and ventilatory systems, consequently reducing an individual’s aerobic scope (i.e. energy available above basic cellular requirements) and impacting all higher functions including growth, feeding, swimming and reproduction^26,27^. Several studies have already shown the adverse effects on the aerobic capacity of tropical fish after exposure to water temperatures approaching their upper thermal limits^28,29^, however no research has focused on how individual fitness is compromised at the lower end of their thermal window. Thermal-tolerance limits of individuals are likely to differ among fish species^18^. A recent laboratory investigation of *A. vaigiensis* showed that winter-water temperatures of less than 18 °C result in depressed feeding rates, growth and burst escape ability, potentially compromising this species ability to survive and persist in temperate habitats^22^. Such negative consequences for the feeding and growth of individuals may then prolong their time spent at smaller size classes and ability to escape predators^30,31^, thereby increasing their risk of predation. Understanding inter-specific differences in thermal performance and whether such temperature-mediated limits may also impact the range of tropical reef fish species dispersing in temperate Australia will be vital in understanding species-specific range shift potential and our ability to predict future range shifts.

This study investigated the thermal performance of two species of juvenile tropical damselfish (*A. vaigiensis* and *A. whitleyi*) that recruit into temperate SE Australian rocky reef habitat^16,21^. The southern distribution of *A. whitleyi* is not as extensive as *A. vaigeinsis* which may result from species-specific differences in thermal performance within this cooler water region. To understand the thermal performance of juvenile fishes, growth rate, feeding rate, burst escape behaviour and metabolic rate were determined across temperatures that reflect typical natal coral reef (26 °C) and SE Australia temperate rocky reefs in summer (22 °C) and winter (18 °C). We tested the hypothesis that these performance metrics would be reduced in *A. whitleyi* relative to *A. vaigiensis* at lower water temperatures.

## Results

### Feeding rates

**Figure 1:**
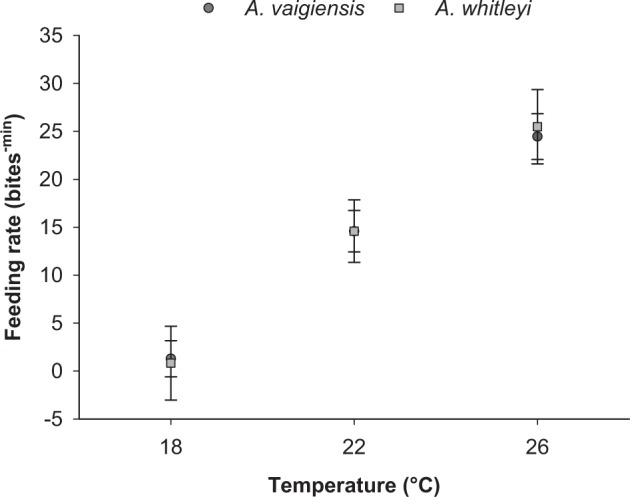
Feeding rate of *A*. *vaigiensis* (n: 18 °C = 21, 22 °C = 16, 26 °C = 13) and *A. whitleyi* (n: 18 °C = 5, 22 °C = 7, 26 °C = 5) maintained at three temperature treatments. Values are least square mean (Mean ± SE) adjusted for the covariate Weight (mean = 0.314 g).

In both species, feeding rates of juveniles increased with water temperature (Fig. 1; F(_2,60_) = 29.627, p < 0.001). Specifically, there was at least a seven fold increase for both species over the temperature range 18 to 26 °C. The greatest change in feeding rate for both species occurred from 18 to 22 °C, but feeding rate differed significantly between all temperature treatments (Tukey’s test for all treatment comparisons; p < 0.001). Feeding rates did not differ between species (F(_1,60_) = 0.006, p = 0.938).

### Growth rates

**Figure 2:**
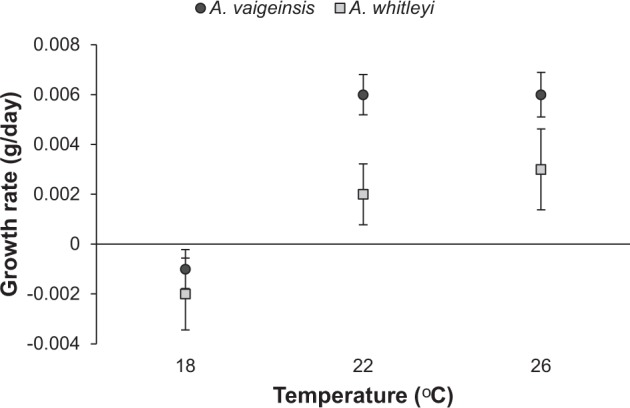
Mean (±SE) growth rate of *A. vaigiensis* (n: 18 °C = 17, 22 °C = 16, 26 °C = 13) and *A. whitleyi* (n: 18 °C = 5, 22 °C = 7, 26 °C = 3) maintained at three temperature treatments. Values are least squares mean (Mean ± SE) adjusted for the covariate Weight (mean = 0.32 g).

Growth rate, calculated as a change in mass per day, was higher at warmer water temperatures of 22 and 26 °C for both species than at 18 °C (Fig. 2; Temperature: F(_2,56_) = 15.424, p < 0.001). Fish maintained at 18 °C lost 0.001 g (approximately 1% loss) per day over the testing period while fish at both 22 and 26 °C increased in weight by approximately 0.004 g (approximately 2.5% gain) per day (22 and 26 °C significantly different from 18 °C, Tukey’s tests, p < 0.05). In addition, *A.vaigiensis* exhibited higher mean growth overall of 0.003 g per day, compared to 0.001 g per day for *A. whitleyi* (F(_1,56_) = 4.738, p = 0.0345). While mean growth rate for *A. vaigiensis* appeared to be similar to *A. whitleyi* at 18 °C and higher at 22 and 26 °C, this apparent difference did not result in a significant interaction (Temperature * Species: F(_1,56_) 3.482, p = 0.67). *A. whitleyi* juveniles were generally smaller at collection, 19.22 mm SL (±0.36 mm SE) compared to 19.79 mm SL for *A. vaigiensis* (±0.09 mm SE).

### Burst swim speed

**Figure 3:**
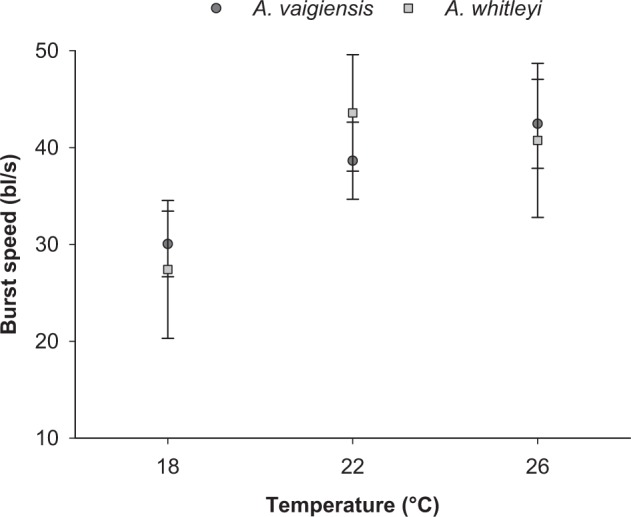
Mean (±SE) burst swimming speed of *A*. *vaigiensis* (n: 18 °C = 22, 22 °C = 16, 26 °C = 12) and *A*. *whitelyi* (n: 18 °C = 5, 22 °C = 7, 26 °C = 4) maintained at three temperature treatments.

Burst swim speed was greater at higher water temperature for both species (Fig. 3; F(_2,60_) = 3.349, p = 0.042). Juvenile *A. whitleyi* exhibited higher burst swim speeds at 22 °C (43.59 bl/s) than 18 °C (27.4) bl/s), but no further increase was seen from 22 to 26 °C (Fig. 3). The significant differences found among temperatures was driven by the 18 °C treatment for both species, which was significantly different from both 22 and 26 °C treatments (Tukey’s tests; 18–22 °C, p = 0.024; 18–26 °C, p = 0.038), while 22 and 26 °C treatments did not differ from each other (Tukey’s test; 22–26 °C, p = 0.935). Burst speed did not differ between the two species, exhibiting similar trends across the 18 to 26 °C temperature range tested (F(_1,60_) = 0.002, p = 0.966; Temperature * Species: F(_2,60_) = 2.296, p = 0.745).

### Aerobic performance

**Figure 4:**
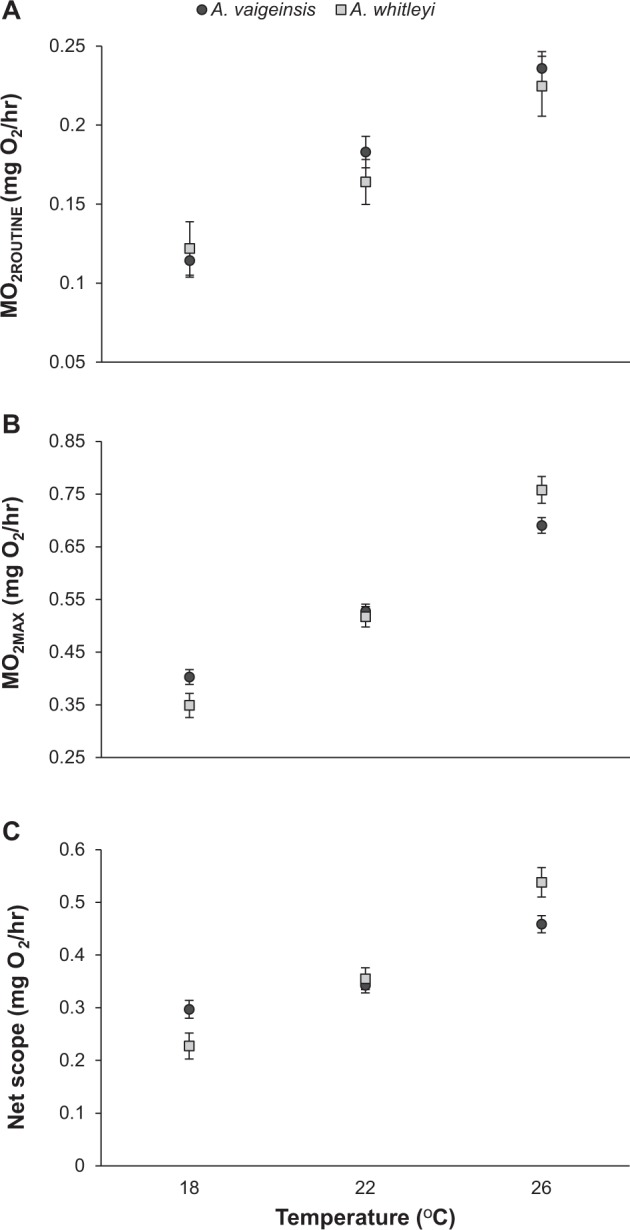
Mean (±SE) (**A**) MO_2ROUTINE_, (**B**) MO_2MAX_ and (**C**) net aerobic scope of *A. vaigiensis* (MO_2ROUTINE_ − n 18 °C = 14, 22 °C = 14, 26 °C = 13; MO_2MAX_ − n 18 °C = 16, 22 °C = 15, 26 °C = 15; net aerobic scope - n 18 °C = 13, 22 °C = 12, 26 °C = 12) and *A. whitleyi* (MO_2ROUTINE_, MO_2MAX_ and net aerobic scope – n: 18 °C = 5, 22 °C = 7, 26 °C = 4) maintained at three temperature treatments. Values are least square means adjusted for the covariate Weight (mean = 0.38 g).

All measured aerobic metabolic attributes increased with water temperature for both species (Fig. 4; MO_2ROUTINE_: F(_2,49_) = 19.35, p < 0.001; MO_2MAX_: F(_2,48_) = 60.101, p < 0.001; Net Scope: F(_2,46_) = 27.579, p < 0.001). MO_2ROUTINE_ increased approximately 0.014 mg/hr per degree of warming, with each temperature treatment found to be significantly different (Fig. 4A; Tukey’s tests, all p < 0.01). MO_2MAX_ increased at a higher rate to MO_2ROUTINE_ of 0.043 mg/hr per degree Celsius and again all temperature treatments were significantly different within a species (Fig. 4B; Tukey’s tests, all p < 0.05). For both MO_2ROUTINE_ and MO_2MAX_ no significant differences were found between species (MO_2ROUTINE_: F(_2,49_) = 0.272, p > 0.05, MO_2MAX_: F(_2,48_) = 0.038, p > 0.05), nor the interaction between species and temperature (MO_2ROUTINE_: F(_2,49_) = 0.384, p > 0.05, MO_2MAX_: F(_2,48_) = 2.088, p > 0.05). However, a significant interaction between species and temperature was observed for aerobic scope (F(_2,46_) = 27.579, p < 0.001). This was driven by higher aerobic scope at 18 °C and lower at 26 °C for *A. vaigiensis* relative to *A. whitleyi* (Fig. 4C).

## Discussion

This study shows that reduced water temperatures approaching critical thermal minima impact key performance attributes of the juvenile stages of two vagrant damselfishes, with performance loss observed below 22 °C. Reductions in feeding, growth, burst-swimming performance and aerobic capacity were observed at 18 °C, compared to 22 °C and 26 °C, for both *A. vaigiensis* and *A. whitleyi*. Some aspects of performance of *A. whitleyi* were also reduced relative to *A. vaigiensis*. Consistent with our hypothesis of reduced performance of *A. whitleyi* relative to *A. vaigiensis* at low temperatures, the reduction in aerobic scope for *A. whitleyi* at 18 °C indicates greater physiological sensitivity at this low temperature compared to *A. vaigiensis*. *A. vaigiensis* also exhibited faster growth than *A. whitleyi* at all temperatures, which may indirectly enhance survival by reducing time spent susceptible to gape-limited predators^30^. Contrary to our hypothesis, however, feeding rate and burst speed (predator escape capacity) were similar between the species. While patterns of feeding rate across temperatures appeared similar between species, with small errors observed around the mean values, the lack of difference in burst speed may have resulted from low numbers of *A. whitleyi*. This species is uncommon in the study region, so sample sizes were relatively low, potentially reducing the power of the experiment to detect the apparent reduction in burst speed for *A. whitleyi* relative to *A. vaigiensis* at lowest temperature (18 °C). However, the significant differences found among temperature treatments suggest that power was significant enough to detect differences at the 0.05 level. With the exception of aerobic scope, the minimal differences found between the species indicate the overall similarities of their physiological and behavioural responses. Regardless, this research compliments our previous understanding of thermal performance and sensitivity of vagrant tropical fish, and emphasises that ocean temperature may need to warm above the performance threshold of 18 °C, rather than the critical thermal minimum, to allow range shifts.

As temperatures approach the lower thermal threshold, the maximal capacity for oxygen delivery declines faster than the basic oxygen requirements, thus limiting aerobic capacity^25,27,32^. These thermal minima temperatures also induce the destabilisation of proteins and enzymes that are essential for performance^25^. Aerobic scope, and the associated MO_2ROUTINE_ and MO_2MAX_, showed high sensitivity to temperature change with significant differences between all testing temperatures. Interestingly, we found that *A. vaigiensis* maintained a slightly higher aerobic scope than *A. whitleyi* at 18 °C; conversely *A. whitleyi* exhibited slightly higher aerobic scope at 26 °C. Despite only small differences between the species’ aerobic scope, minor changes to aerobic capacity can impact individuals and ultimately populations through energetic trade-offs in behavioural and physical performance^27^. However, the observed differences between species are small in comparison to the overall reductions observed in relation to water temperature. Over the 8 °C temperature range tested aerobic scope was reduced by approximately 25%. This reduction in aerobic scope is expected to correlate to a reduction in energy available for higher level functions including growth^27,32^, and may indirectly explain why *A. whitleyi* distribution is limited at lower latitudes in NSW^16^. We did observe some differences in growth that matched trends in reduced aerobic capacity from 22 to 18 °C. However, the lack of corresponding differences in growth between 26 and 22 °C, which saw substantial reductions in aerobic capacity, suggests that while we may simplistically expect a direct relationship between energetic costs, energy intake and linear growth there are numerous other aspects of morphological development that we have not measured here that would be energy consumptive.

Similar trends with temperature were observed for burst escape performance, feeding rate and growth in both species, with the largest changes observed between 18–22 °C, rather than 22–26 °C. The broad range of traits affected potentially indicates that a number of critical physiological processes are impacted at water temperatures less than 22 °C. This result is not unexpected, since the average minimum winter water temperature on natal corals reefs in the southern GBR is 21 °C^22^, and these species may not have evolved to tolerate cooler waters. The observed reductions in performance at 18 °C matches with field observations of seasonal mortality during winter (June-August) in NSW coastal waters, which on average is between 15–17 °C^18,22^. In combination, these results suggest SE Australia waters would need to warm above 18 °C before range shifts may be thermally possible, and likely higher for substantial overwintering and establishment of populations.

While we observed differences in burst speed among water temperatures, it was the least sensitive performance metric to changes in water temperature. This is probably due to the fact that at high velocity swimming, white anaerobic muscles provide the power needed for burst speed^33^, whereas the other performance traits measured were powered by the aerobic systems. While burst speed was not as sensitive to temperature change, even small differences in response speed could have severe impacts on survival^34^. Although no significant differences were detected between species in the current study, at 18 °C *A. vaigiensis* juveniles swam on average 7 body lengths greater than *A. whitelyi* juveniles, which, if confirmed as a difference in future studies employing greater replication, may have implications for survival and population establishment in temperate habitats. Even a slight species-specific difference in escape distance may be the determinant of predator avoidance and ultimately survival. There are a number of other components of the burst escape response that were not measured in the study, specifically directionality, C-start latency and formation and Mauthner neurons (M-cell) excitability^31,35^. Action-potential in one M-cell triggers intracellular responses that initiate a fast-body bend (C-start), or escape response, away from an aversive stimulus^36^. While not confirmed in the current study, relative burst escape performance warrants further investigation, as a potential driver of differences in range shift potential among vagrant fishes.

The similarities in thermal performance between *A. vaigiensis* and *A. whitleyi* for some traits measured indicate that temperature is not likely the sole driver of variation in juvenile range and overwintering likelihood between the two species. Instead, ecological and biophysical interactions with temperature and other environmental factors may govern distribution and overwinter survival^21^. For example, predation acts as a major driver of population size and is often size-dependent, with higher predation mortality in smaller-size classes^37^. It has been observed in previous research that in expatriated populations of vagrant damselfish, *A. whitleyi* are generally smaller that *A. vaigiensis*^38^, potentially resulting in more predation of *A. whitleyi* aggregations. Differences in survival between species may also result from Allee effects^39^, with the greater abundance of *A. vaigiensis* compared to *A. whitleyi in situ*^16^ potentially contributing to overwintering differences. If population fitness and growth are density-dependent, then the smaller aggregations of *A. whitleyi* will exhibit reduced growth and spend greater periods of time at vulnerable size classes, increasing the likelihood of predator-induced mortality^21^.

The current settlement patterns of tropical fish vagrants into temperate SE Australia are correlated with rises in sea surface temperatures, and results obtained in this study indicate that winter temperatures greater than 18 °C could facilitate range shifting of both *A. vaigiensis* and *A. whitleyi*. Our findings build on previous studies by highlighting that performance loss can be observed at 18 °C, prior to critical thermal minimum temperatures around 16.5–17.5 °C^18^. We have shown some support for the expectation that *A. whitleyi* would be more thermally sensitive to cooler temperatures than *A. vaigiensis*, based on a more restricted poleward distribution. However, similarities observed for some traits, along with known interactions between environmental and ecological limiters of population size, suggest that future persistence will have more complex mechanisms than thermal performance alone, including aspects unmeasured here such as predation and/or competition, Allee effects and differences in recruit supply^16,21,22^. Extrinsic factors such as temperature and strengthening poleward ocean currents will likely increase the influx and range shift potential of tropical species into temperate marine ecosystems, and intrinsic factors will likely determine which species will establish viable populations that may influence the ecological dynamics of these systems, including competition and predation^15^. Further investigation of the lower end (18–22 °C) of tropical vagrants’ thermal minimums is essential for predicting future range shifts as anthropogenic-induced climate change alters the physical variables of marine ecosystems.

## Materials and Methods

### Experimental design

Juvenile *A. vaigiensis* (n = 50; standard length (SL) range 13–35 mm) and *A. whitleyi* (n = 17; SL range 16–31 mm) were collected in several batches between the 27^th^ of February and the 2^nd^ of May, 2013, from rocky reef habitats at 3 locations along the Sydney coast (Collins 33°48′30″S, 151°17′25″E, Freshwater 33°46′54″S, 151°17′39″E, and North Narrabeen 33°42′08″S, 151°18′25″E headlands), using an anaesthetic (clove oil, ethanol and sea water mixture) and hand net^40^. For *A. vaigiensis*, batch 1 was caught on the 27^th^ of February, batch on the 26^th^ of March and batch 3 on the 9^th^ of April. For *A. whitleyi*, batch 1 was caught on the 19^th^ of March, batch 2 on the 26^th^ of March and batch 3 on the 14^th^ of April. Fish were transported live within 3 hours and randomly assigned to 1 of 3 temperature-controlled aquariums at the University of Technology Sydney. Individual fish were placed in opaque-walled 14 L aquaria that were maintained at the ambient ocean temperature from the collection location (~22 °C). Over the course of 5 days, 1 tank was maintained at 22 °C, whereas the others were either lowered or increased in temperature of approximately 1 °C per day depending on their intended treatment tank. Following a habituation period of 5 days, individual fish were assigned randomly into the 3 temperature treatments: 26 °C, 22 °C and 18 °C. Overall for *A. vaigeinsis* there were 22 assigned to 18 °C, 16 to22 °C and 13 to 26 °C; and for *A. whitleyi* 5 assigned to 18 °C, 7 to 22 °C and 5 to 26 °C. Treatments were chosen to mimic the different environmental conditions that juvenile *Abudefduf* species may experience during their life history. The 26 °C treatment mimicked the average early summer temperatures experienced on the Southern Great Barrier Reef (GBR), the most likely source of tropical vagrants in temperate SE Australia, and parallels the irregular maximum sea surface temperatures of SE Australia during the recruitment period^35^. The summer temperature in the coastal waters of Sydney is approximately 22 °C^41^, corresponding to arrival and settlement of both *Abudefduf* species. The 18 °C temperature treatment mirrored the average water temperature at the end of the recruiting season in Sydney (May-June), and approached the lower critical thermal limit of approximately 17 °C previously identified for *Abudefduf* species^22^. Temperature treatments took 5 days to reach the desired set points, averaging a change of 0.8 °C daily for the 18 and 26 °C treatments. Water temperature was maintained by 25 W aquarium heaters in each tank and remained at ±0.5 °C of the treatment for 23 days. The tanks maintained at 18 °C were controlled by the thermostatted air-conditioning system, but were still supplied with non-operative heaters, to mimic secondary treatment effects. Tank temperatures were taken daily, and ammonia and pH tests were performed within the first and last weeks of the treatment period. To provide shelter for all experimental animals each tank also contained a 10 cm section of PVC tubing (6 cm diameter).

All fish were fed *ad-libitum* with 7 Spectrum marine fish food pellets 3 times daily. Uneaten food and faeces were siphoned off daily (prior to first feeding), simultaneously removing approximately 1–2 L of water, which was replaced with clean water (15–20% of the water from each tank).

### Feeding rates

To examine feeding rate under standard hunger conditions all fish were starved for 12 hours prior to being supplied with an excess amount of food (following^16^). The number of bites taken by fish for 60 s post-feeding was counted by observation. All feeding assessments were conducted during the second week of the experiment.

### Growth

At the commencement of the experiment, once fish had reached acclimation temperature, all individuals had their standard length (SL) measured to the nearest 0.5 mm, total length (TL) measured to the nearest 0.5 mm (both measured with a mm ruler) and wet weight (WW) measured to the nearest 0.01 g. These were also measured at the completion of the experiment as described above. Somatic growth was assessed by calculating growth rate as a change in WW over the experimental period.

### Burst swimming speed

Predation is the major cause of mortality in marine fish populations; therefore the burst swimming speed used for the escape response by a fish is critical for survival^42^. The burst response of fish was determined by video analysis during the second week, 5–7 days after commencing temperature treatments. Fish were transferred individually to a narrow opaque-walled glass aquarium (10 cm wide, 50 cm long 30 cm tall) that had a gridded background (0.5 cm grids) with water that was maintained within 0.5 °C of their treatment temperature. The sides and back of the tank were blacked out to prevent external behaviour disturbance, and a Panasonic LUMIX DMC-FT4 waterproof digital camera was mounted in front of the tank. The methodology followed the protocols used in^22^, where individuals were startled using a dropped weight within the tank. After a 5 minute acclimation period, fish were recorded on video for 1 minute to quantify premature startle response before the experiment started. After this minute elapsed, fish were startled by a magnetic weight, which was released in the far side of the tank, and all behaviours recorded for a further minute. Videos were recorded at 24 frames per second, which has been proven adequate in assessing burst swimming speed in similar species^22,24^. Videos were analysed using Tracker 4.80 Video Analysis and Modelling Tool programs, which allowed videos to be slowed down to 0.04 frames per second. The greatest distance moved between 2 adjacent frames (approximately 0.04 s elapsed time) after the weight was released was taken as the maximum burst swimming speed of a fish in that trial. This measurement was originally taken in mm/s and then converted to body lengths per second (bl/s) by dividing the mm/s travelled by the TL of the fish to account for the effect of body length on distance moved.

### Aerobic performance

Metabolic attributes, estimated from oxygen consumption, of *A. vaigiensis* and *A. whitleyi* were measured with static daytime respirometry 19 days after commencing temperature treatments, between 08:00–12:00^43,44^. Static measures produce consistent and reliable measures of MO_2ROUTINE_ for damselfish species, likely due to constant pectoral fin movement^45,46^. Fish were starved for 12–24 h prior to metabolic testing to remove the effects of digestion on oxygen consumption. To measure MO_2ROUTINE_, fish were transferred to respirometers (295 mL opaque plastic jar with a 60 mm diameter, covered with black tape except for the bottom), which were submerged in a temperature controlled aquarium that mirrored their temperature treatment. Rate of oxygen usage was measured using the PreSens Fibox contactless sensor system (Precision Sensing GmbH). At the commencement of testing, flow to the respirometers was ceased and oxygen levels in each chamber were measured over a 20 min period. At the conclusion of MO_2ROUTINE_ testing, the respirometers were opened to once again allow oxygenated water flow through the chamber. A pilot study determined that 2 hrs habituation in the respirometers was sufficient time for a fish to recover from handling (recorded lowest rates of oxygen consumption). This recovery time was similar to that found for other damselfish species^45,47^. To measure MO_2MAX_ a swim respirometry chamber was used; consisting of a Perspex cylinder (150 mm inner diameter and 330 mL volume), below which a magnetic plate was placed to drive the stirring bar in the respirometer, creating a current, which the fish swam against^28,43,48^. The speed of the water was set so that the fish could maintain a sustained and consistent speed that was close to burst (which is sporadic). MO_2MAX_ was calculated from 5 minutes of oxygen usage and was derived using the slope of the linear regression between oxygen availability and time, per fish. Both MO_2ROUTINE_ and MO_2MAX_ were calculated in mg O_2_ h^−1^, and the difference between these attributes was calculated as net aerobic scope (MO_2MAX_ − MO_2ROUTINE_).

### Statistical analysis

Potential differences in burst speed (bl/s) depending on temperature (fixed factor) and species (fixed factor) were tested using a factorial Analysis of Variance (ANOVA). In the case of feeding rates, growth rates (change in WW) and all metabolic variables (MO_2ROUTINE_, MO_2MAX_ and Net Scope), individual size influenced the trait response (p < 0.05 for all results). Consequently, factorial GLMs were run with temperature and species as fixed factors, and mass (WW) as a covariate to account for the effect on the variable of interest. In the case of growth rate and feeding rate the initial WW of fish was the mass covariate. For all metabolic variables WW at the time of measurement was used as the covariate. To adhere to the linearity assumption, WW and the metabolic response variable were ln(x + 1) transformed prior to running analyses. The relationship between mass and the factor of interest (growth, feeding and respiration) was consistent across the temperatures (all p < 0.05). Least-square means adjusted for the effect of the covariate are presented throughout. Where results indicated significant differences, Tukey’s *post hoc* tests were run to identify differences between levels within a factor. Data were graphically inspected for normality and homogeneity of variance was examined using Levene’s test. Data conformed in all cases. All statistical analyses were completed using IBM SPSS^®^ Statistics v23.

### Compliance with ethical standards

Ethics approval for this study was obtained from the UTS Animal Care and Ethics (Approval Number: UTS ACEC 2012-433A). All applicable international, national, and/or institutional guidelines for the care and use of animal were followed.

## Supplementary information

Supplementary Dataset 1

## Data Availability

The datasets generated during and/or analysed during the current study are available from the corresponding author on reasonable request.
